# Genomics, Transcriptomics, and Proteomics of SSV1 and Related Fusellovirus: A Minireview

**DOI:** 10.3390/v14102082

**Published:** 2022-09-20

**Authors:** Martina Aulitto, Laura Martinez-Alvarez, Salvatore Fusco, Qunxin She, Simonetta Bartolucci, Xu Peng, Patrizia Contursi

**Affiliations:** 1 Dipartimento di Biologia, University of Naples Federico II, 80126 Naples, Italy; 2 Lawrence Berkeley National Laboratory, Biological Systems and Engineering Division, Berkeley, CA 94720, USA; 3 Archaea Centre, Department of Biology, University of Copenhagen, DK-1165 Copenhagen, Denmark; 4 Biochemistry and Industrial Biotechnology Laboratory, Department of Biotechnology, University of Verona, 37134 Verona, Italy; 5 CRISPR and Archaea Biology Research Center, State Key Laboratory of Microbial Technology, Microbial Technology Institute, Shandong University, Qingdao 250100, China; 6 BAT Center—Interuniversity Center for Studies on Bioinspired Agro-Environmental Technology, University of Naples Federico II, 80055 Naples, Italy; 7 Task Force on Microbiome Studies, University of Naples Federico II, 80126 Naples, Italy

**Keywords:** *Saccharolobus*, SSV1, genomic, transcriptomic, proteomic

## Abstract

*Saccharolobus* spindle-shaped virus 1 (SSV1) was one of the first viruses identified in the archaeal kingdom. Originally isolated from a Japanese species of *Saccharolobus* back in 1984, it has been extensively used as a model system for genomic, transcriptomic, and proteomic studies, as well as to unveil the molecular mechanisms governing the host–virus interaction. The purpose of this mini review is to supply a compendium of four decades of research on the SSV1 virus.

## 1. Introduction

**Figure 1:**
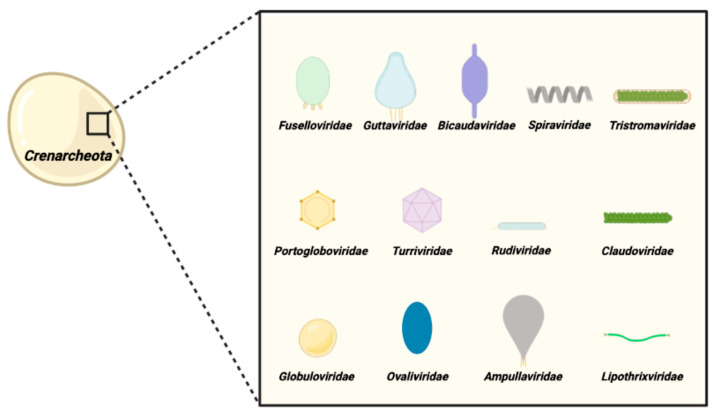
**Representation of viruses infecting Creanarcheota.** The schematic morphologies of virions are shown above the corresponding family names.

With some exceptions, archaeal viruses are genomically and morphologically unique since they are evolutionarily unrelated to viruses infecting bacteria and eukarya, thus representing one of the least understood part of the virosphere [1,2,3]. Indeed, most of them harbor many orphan open reading frames (ORFs) with little or no similarity to known sequences in the public databases [4]. Most of the known archaeal viruses infect members of the phylum *Crenarchaeota*, and those unique to hyperthermophilic *Archaea* belong to 13 families, i.e., *Turriviridae, Portogloboviridae, Rudiviridae*, *Clavaviridae*, *Lipothrixviridae, Tristromaviridae*, *Globuloviridae*, *Ovaliviridae*, *Guttaviridae*, *Spiraviridae*, *Ampullaviridae, Fuselloviridae,* and *Bicaudaviridae* [5]. Some morphologies are thus far exclusive to archaeal viruses, including the bottle (*Ampullaviridae*), the droplet (*Guttaviridae*), and the spindle or lemon shape (*Fuselloviridae*). Among these latter ones, SSVs (*Saccharolobus*-*ex Sulfolobus*-spindle virus) were the first discovered and one of the best studied archaeal viral families (Figure 1) [6].

SSVs are abundant in environments characterized by extreme chemo-physical conditions such as high temperature (>70 °C) and low pH (<4), where their hosts, i.e., mainly *Sulfolobus* spp. (recently renamed *Saccharolobus* [7]) and its close relatives, thrive [8]. SSVs contain circular double-stranded DNA genomes that vary in size between 14 and 17 kbp and are found as episomal DNA, integrated in the host genome as proviruses, or packaged as fully assembled virions [6,8]. Their size is approximately 90 nm by 60 nm with short tail fibers extending from the end of the major axis. A total of 50 SSV representatives have been deposited into the NCBI (20 June 2022, Supplementary File S1); nevertheless, the majority of them have been poorly characterized, apart from SSV2, SSV8, SSV9, and SSV22, for which sporadic biochemical and physiological studies are reported [9,10,11,12,13,14].

Among SSVs, *Saccharolobus* spindle-shaped virus 1 (SSV1) is the best-understood member of the *Fuselloviridae* and represents one of the model archaeal viruses for physiological, genetic, transcriptional, and virus–host interaction studies [13,15,16,17,18]. SSV1 was isolated from its natural host (*Saccharolobus shibatae* strain B12) at a geothermal spring in Beppu (Japan). Initially, SSV1 was named SAV1 because of the erroneous species annotation of its host that was at recognized as *Saccharolobus acidocaldarius* B12 [17,19,20,21]. SSV1 can replicate in few other hosts, including a strain of *S. solfataricus* isolated from the solfataric field of Pisciarelli near Naples (Italy) [12]. Upon infection, a copy of the SSV1 genome (i.e., a double-stranded DNA molecule of 15,465 bp) integrates into the host chromosome at an arginyl-tRNA gene by means of a virus-encoded integrase (D355) [22]. Besides the integrated form (pro-virus), about five copies per cell of SSV1 DNA are present in host cells as episomal DNA [23]. As reported by initial transcription analyses, upon UV irradiation, a well-coordinated temporal expression of the SSV1 transcripts was revealed, similarly to what has been reported for many bacteriophages and eukaryotic viruses [24,25]. Sequence analyses of SSV1 transcriptional start sites (TSSs) led to the discovery of archaeal promoter elements such as the TATA-box-like hexanucleotide sequence as well as the nearby TGA consensus trinucleotide sequence [24,26]. These two conserved motifs resemble those recognized by the eukaryotic RNA polymerase II [26], thus implying a similar functional recognition mechanism of the archaeal RNA polymerase [26,27]. Most proteins encoded by SSV1 have little sequence similarities with other characterized proteins in public databases; consequently, their function has been challenging to infer through homology analysis. Notorious exceptions are some of the SSV1 structural proteins (VP1, VP2, and VP3) and the integrase [22,28].

The purpose of this mini-review is to provide a concise overview of all the work performed on SSV1 including genomic, transcriptomic, and proteomic studies; virus–host interaction; and co-evolution.

## 2. Genomic Analysis

### 2.1. The Fusellovirus Core Genome

**Figure 2:**
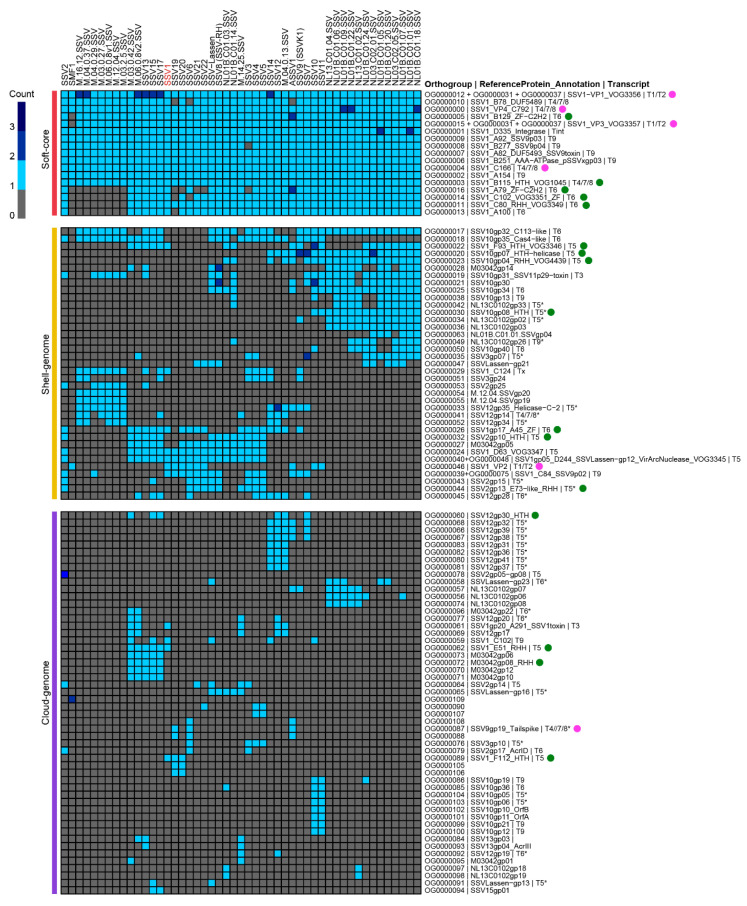
**The pangenome of the *Fuselloviridae*.** Presence and absence heatmap of clusters of homologous proteins in 49 fuselloviruses. Genes present in > 80% of the strains were labeled as part of the soft-core genome (red line), genes present in >20% of the genomes are part of the shell-genome (yellow line), and the rest are considered the cloud-genome (purple line). Singletons are not shown. Pink circles indicate virion proteins, and green circles indicate putative transcriptional regulators. Asterisks (*) indicate that location of the reference gene within the corresponding genome is putative.

Previous efforts to identify the core genes of fuselloviruses used 9–11 genomes and identified a set of 12–13 genes conserved in members of this family [16,29,30]. However, there are 50 fusellovirus sequences currently available in the NCBI, and we set up to determine if the earlier defined set of core genes accurately reflected gene conservation of the expanded family. The genomic sequences of the *Fuselloviridae* available in the NCBI (June 2022) were downloaded and clustered to 98% nucleotide identity and used for further analysis (49 genomes). A total of 1595 ORFs were retrieved from these genomes, and OrthoFinder v2.5.4 was used to define clusters of homologous proteins [31,32] (see Supplementary Files S1–S4). This resulted in 109 clusters of two or more protein sequences (orthogroups) covering 1528 (96%) of the ORFs analyzed, plus 67 singletons (proteins with no homologs in other fuselloviruses). A heatmap of the distribution of orthogroups in the fuselloviruses is shown in Figure 2. With the result of our analysis, we revisited the definition of the *Fuselloviridae* core genome. We propose 17 genes as the soft-core of the fuselloviruses, which are present in over 80% of the genomes (Figure 2, red line). The shell genome (yellow line) includes genes present in >20% of the genomes, and the variable genome in Figure 2 (purple line) contains genes with more than two occurrences in the dataset. For simplicity, singletons are not shown.

### 2.2. Location of Core Genes

The fuselloviruses have a similar genome organization where genes are clustered into 10–12 transcriptional units and homologous genes are encoded usually in the same transcript across different genomes [11,24,25,33]. Transcripts T_4/7/8_, T_9_, and T_1/2_ contain most of the core-genome ORFs, while the shell and variable portions of the genome are identifiable in transcripts T_5_ and T_6_. The latter are highly flexible regions in the fuselloviruses and contain most of the non-essential genes identified in previous functional genomics studies [11,34,35]. Among the genes in the soft-core genome, nine are present in all genomes and constitute the absolute-core (VP1, VP4, D355-integrase, A82, A92, B251-AAA ATPase, C166, A154, and B115), while four genes are present in over 90% of the genomes (VP3, B129, B277, and B78). The four remaining soft-core genes (A79, C102, C80, and A100), despite being missing from a small subgroup of viruses isolated primarily from Russia, were thus far considered as part of the absolute-core [29,30].

### 2.3. Functional Annotation of Core Genes

The 13 formerly defined core genes [14,29,30] were kept in our analysis as part of the soft-core, and we incorporated four additional genes (A92, VP3, C102, and A100). Core genes VP1, VP3, VP4, and C166 play a structural role [28,36,37,38], the integrase D355 serves virus integration into the genome [22], and proteins B251 an ATPase with similarity to the bacterial DnaA [39] and A154 have been implicated in replication and packaging of the genome. Indeed, B251 and A154 are also encoded by the satellite virus pSSVx, which replicates during infection of an SSV2 helper [40]. Although homologs of the tail-spike VP4 are present in all the SSV sequences analyzed, six genomes do not have a full-length version of the protein and it has been questioned if this truncated version is able to complement the structural function of the full-length VP4 [30]. Three of these genomes (SSV NL101B.C01.09, NL101B.C01.18, and NL101B.C01.22) harbor two VP4 homologs that seem to be the result of the full protein split into its N- and C-terminal domains. For the remaining three (SSV6, SSV19, and ASSV1), it was recently reported that the short VP4 homolog serves as an adaptor protein for the tail, while another protein (belonging to OG0000087) has the same genomic location as VP4 and serves as the tail-spike [38]. A truncated VP4 and the OG0000087 tail-spike are associated with viruses that show a pleomorphic morphology [30]. Since genome organization seems conserved in fuselloviruses and given that transcripts T_4/7/8_ and T_1/2_ appear to encode for structural proteins, we predict B78 to be also a virion protein as it has two transmembrane helices (similarly to VP1, VP3, VP4, and C166, which also contain transmembrane helices). Intriguingly, homologs of this protein are missing from SSV6, SSV19, and ASSV1, which have a different tail structure [38]. Interestingly, the absolute core gene B115, which is a putative transcriptional regulator, is encoded in T_4/7/8_ and may be involved in the regulation of structural gene expression. The core protein A82 was recently demonstrated to act as a toxin that kills uninfected cells [41]. The corresponding gene lies in the same region of all SSV genomes, downstream of the genes encoding capsid proteins VP1 and VP3, in the proximity of the core proteins A92 and B277 and of the non-core protein C84. The role of these accompanying proteins in the infection cycle remains to be elucidated and may not be related to the replicative advantage conferred by the toxin [41]. Interestingly, the work of DeWerff et al. [41] identified additional toxins (OG0000019 represented by SSV11p29 and OG0000061 represented by A291), which are not part of the core genome of the *Fuselloviridae.*

### 2.4. The Fusellovirirus Pangenome Contains Multiple Transcriptional Regulators

It is noteworthy that around one-third of the genes in the core genome (5/17) are putative transcriptional regulators (A79, C80, C102, B115, and B129), and this holds true also for several genes of the shell- and cloud-genomes (Figure 2, green circles). These transcriptional factors have not been experimentally characterized, although a couple of studies address the role of regulators that do not belong to the core genes [33,42,43,44,45,46,47]. C80 is homologous to the host transcriptional regulator aCcr-1 (SSO_RS11690 in *S. solfataricus* P2), a global cell cycle regulator in the Sulfolobales. Homologs of aCcr-1 are widespread in viruses infecting Sulfolobales and are thought to benefit virus replication by driving the cell into the S-phase of the cycle where DNA synthesis occurs. This ensures the presence of proteins and resources required for viral genome replication, which depends on the cellular machinery [47]. However, while the C80 homologs of SSV19–22 and ASSV1 have high sequence similarity to aCcr-1, the other members of the C80 cluster are divergent, cluster separately to aCcr-1 and consist of two different RHH-domains linked together, implying a functional difference [47]. The prevailing presence of transcriptional factors in fuselloviruses suggests that transcriptional regulation of viral gene expression is critical to their infection, as illustrated by F55 (see below), a protein unique to SSV1 involved in the control of early gene expression after UV induction of viral replication [33,45]. 

Table 1 summarizes the 10 putative transcriptional factors in SSV1. The remaining core proteins C166, B78, and A100 have no annotation and their functions remain to be investigated.

**Figure 3:**
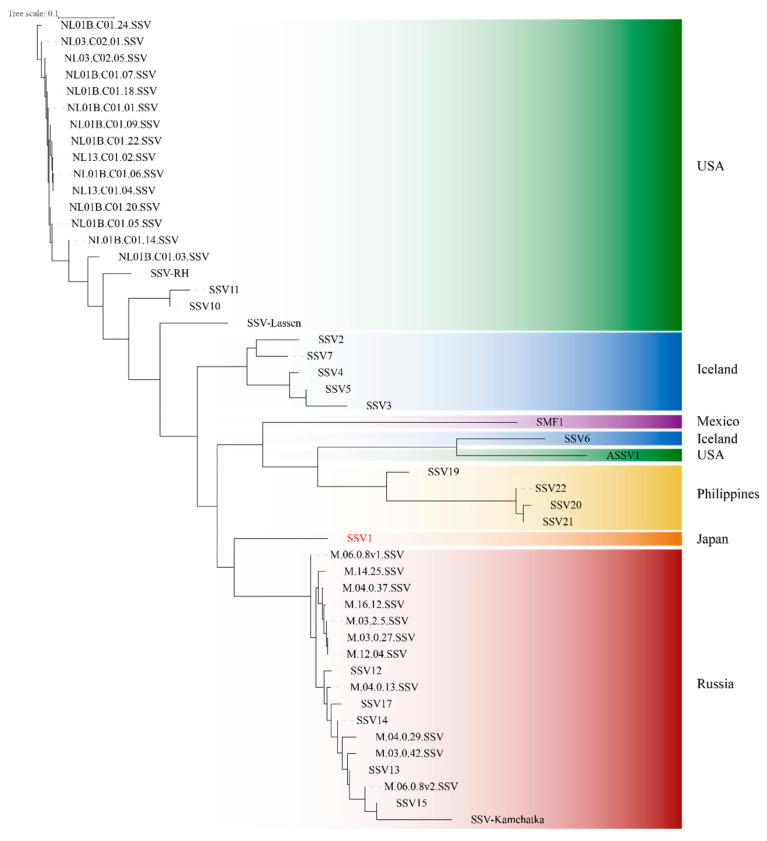
**Phylogeny of the *Fuselloviridae*.** The phylogeny of the 49 fusellovirus sequences was obtained with OrthoFinder, which uses the predicted orthogroups to infer a tree using STAG and roots the tree with STRIDE [31,50,51]. Viruses cluster according to their geographic location, as shown by the color code.

The genome of SSV1 contains a high number of singletons in comparison to the other members of the family (7/35, F55, A132, B49, E96, E178, F92, E54). This is among the highest number of singletons (only surpassed by SMF1, which has 10), as the majority of fuselloviral members (70% of the genomes) possess 0–1 singletons. Most of these unique proteins are encoded in transcript T5, which, together with T6, contains the bulk of the variable gene pool of fuselloviruses. Overall, the core genome of the *Fuselloviridae* appears well defined, and the incorporation of new genomes did not dramatically change the set of core genes. Genome organization is also conserved and maintained in transcriptional units of genes participating in similar processes (e.g., virion structure and assembly). Nevertheless, the role of most of the viral proteins remains to be elucidated. Finally, a phylogenetic tree of the *Fuselloviridae* family is shown in Figure 3. Viruses cluster according to their geographic location, a pattern that has been observed previously for the *Fuselloviridae* and the *Rudiviridae* [29] and for genes of other viruses [49].

## 3. Transcriptomic Analysis

### 3.1. SSV1 Genes Regulation

The analysis of SSV1 transcripts dates back to 1987, when Zillig and coworkers performed a pioneering study on transcription expression in *Archaea* using SSV1 as a model system [24,28]. At that time, it was known that SSV1 was a UV-inducible virus able to infect *S. shibatae* and *S. solfataricus*, exhibiting a unique life cycle that did not imply cell lysis [20]. However, the molecular mechanisms underpinning the switch from lysogeny (carrier state) to the induction state were not known for a long time, as well as those regulating the maintenance of the lysogeny and the reversion to the steady state in the aftermath of UV induction. Moreover, the complex network regulating the host–virus interaction had not yet been elucidated.

### 3.2. SSV1 Genes Expressed in the Carrier State

**Figure 4:**
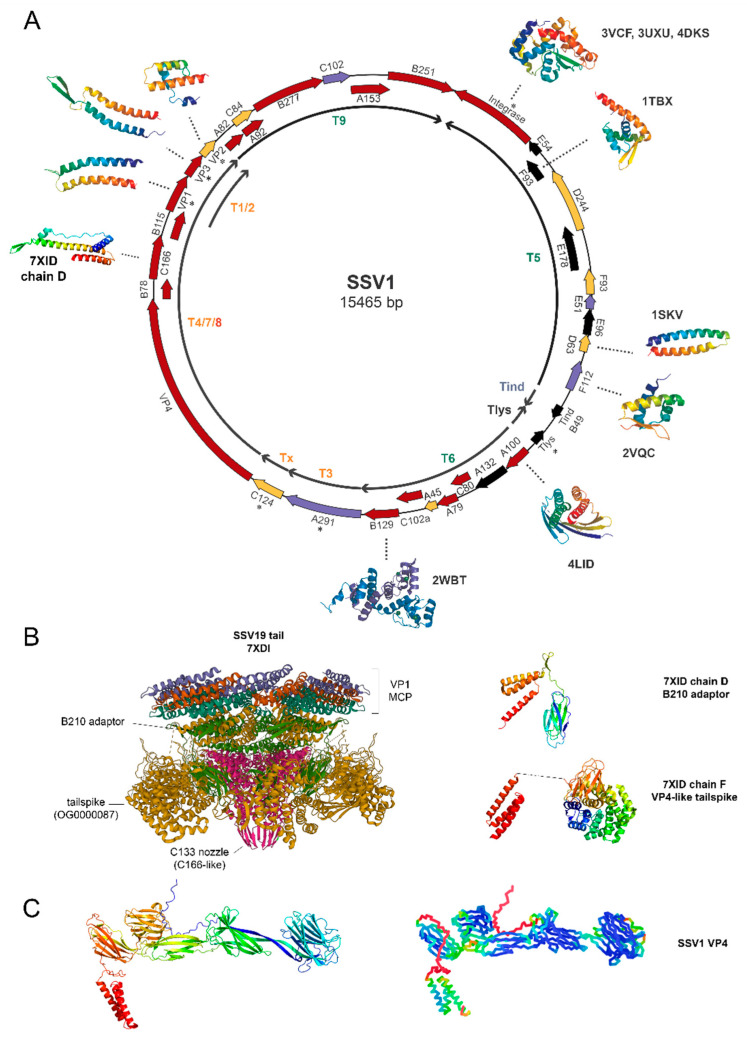
**Genomic map of SSV1.** (**A**) ORFs in the genome of SSV1 are depicted as arrows blocks, and coloring corresponds to their classification as part of the soft-core (red), cloud (yellow), or variable (purple) genome. Proteins expressed during lysogeny are marked with an asterisk (*). Inner arrows represent the transcripts of SSV1, and the font color indicates their classification according to their transcriptional profile into UV-induced (blue), early (green), late (orange), and late-extended (red) transcripts. Tlys is transcribed in the lysogenic state. Crystallographic structures available for SSV1 open-reading frames are depicted for the integrase D355, F93, D63, F112, A100, B129, and C166 (from the SSV19 homolog C131). The structures of VP1, VP2, VP3 (**A**), and VP4 (**C**) were modelled using AlphaFold2 [53]. (**B**) Cryo-electron micrograph of the SSV19 tail (7XID, [38]). The atomic models of the B210 adaptor and the VP4-like tail-spike are shown to the right. (**C**) Model structure of SSV1 VP4. Left: atomic model of SSV1 VP4. Right: model colored according to the confidence measure of the prediction (pLDDT) on a scale from 0 to 100, where blue indicates high confidence and red indicates low confidence.

The first studies on transcriptional mapping of SSV1 highlighted the presence of 11 transcripts that started from 7 mapped promoter sites and covered almost the whole genome, which includes 35 open reading frames (ORFs) [17,24]. Constitutive messengers expressed once the lysogenic state has been established are T1/T2, T3, and Tx, encoding *vp1-2-3, a291* (a putative toxin) and *c124* genes [23,25] (Figure 4). A transcriptional activity has been detected also for *d335*, encoding the SSV1 integrase. Then, the carrier state of SSV1 is sustained by the expression of a few genes, among which only those encoding the structural capsid proteins (VP1, VP2, and VP3) [34,52] and the integrase have been functionally characterized [22]. Moreover, a recent study has revealed that most of the lysogenic genes (*vp1, vp2, d355,* and *a291*) are apparently essential for SSV1 infectivity, except for *c124* and *vp3* [16]. Subsequently, a transcriptomic analysis revealed an additional mRNA (named T_lys_) in uninduced cells. T_lys_ encodes a 55-amino-acid protein (F55) that is involved in the repression of the early UV-inducible genes (see below) in the absence of UV irradiation [33]. Genetic functional analysis on *f55* has shown that this gene is not required to produce infectious virus particles. Rather, it seems that ΔF55 mutants exhibit a constant expression of early gene products because of the absence of the repressive action of F55 on the early promoters [16].

### 3.3. SSV1 Genes Expressed upon UV Induction

The study of the SSV1 life cycle in the aftermath of UV induction revealed the very early expression of T_ind_ transcript (Figure 4) whose role is still murky [16,25]. Initially, it was hypothesized that this transcript triggered the initiation of replication, thus pointing to the T_ind_ surrounding region as the possible replication origin of SSV1 [24]. Subsequently, the identification of a 49 aa long protein (B49) within T_ind_ messenger suggested an alternative role in switching on the viral transcription, either directly or indirectly [19,33]. Indeed, secondary structure prediction indicates that B49 might bear a helix-turn-helix motif, thus indicating a possible role as transcription factor. However, expression trials of B49 gene in *E. coli* and in *S. solfataricus* were unsuccessful (unpublished data), thus casting doubts about the true translation of T_ind_ into a functional B49 protein. Although direct evidence of the T_ind_ role is still lacking, the fact that its expression extensively increased in the timeframe following the UV induction and preceding the onset of SSV1 replication suggested that it plays a role in UV-induced replication either as a transcript and/or as a protein product [24,54]. Finally, mutational studies demonstrated that deletions of T_ind_ regions are indeed tolerated, thus showing that this transcript is dispensable for the infectivity of virus particles in the absence of UV stimulus [16] (Figure 4). Upon UV induction, the very early expression of T_ind_ transcript is followed by the transcription of the early (T_5_, T_6_, and T_9_), late (T_1/2_, T_3_, T_x_, and T_4/7_), and late-extended (T_4/7/8_) RNAs [24,25]. This cascade of events led, in turn, to the induction of the SSV1 genome replication and eventually to a steep increase in the viral titer. The two subsets of T1/T2 and T4/T7/T8 transcripts originated from a common promoter located upstream of T1 and T4, respectively, and terminator read-through resulted in the formation of more than one RNA species [17,26] (Figure 4).

### 3.4. Promoters and Terminators of SSV1

Mapping of the transcriptional start sites (TSSs) of the SSV1 transcripts has allowed the discovery of archaeal promoter elements such as the TATA-box-like hexanucleotide sequence (box A, TTTAAA) and the nearby TGA consensus trinucleotide sequence (later identified as the BRE, B recognition element sequence) located about at −23 and −28 nucleotides upstream of the TSS [24,26]. Moreover, transcription termination signals of SSV1 messengers were associated to the presence of a TTTTTYT conserved motif. All these consensus sequences resembled those found in eukaryotic promoters and terminators recognized by RNA polymerase II. This evidence mirrored a similar functional recognition mechanism of the archaeal RNA pol as confirmed by its homology to the eukaryotic RNA polymerase [26,27].

### 3.5. SSV1 Transcription Regulation

The transcriptional regulation of SSV1 genes upon irradiation is quite complex and implies a combination of different mechanisms including mRNA degradation [24], early transcriptional termination/antitermination processes [26], up- and downregulation [20], and possible cooperation of some of these. For instance, terminator read-though took place for terminators of transcripts T7 and T4 that were included in a polycistronic T8 messenger upon UV irradiation. Likewise, T2 transcript was included in T1 upon UV exposure [26]. A similar mechanism is employed by pSSVx, thus suggesting that viruses can discard the regulative role of transcriptional sequences under conditions that trigger virus replication [10]. Whilst most of the mechanisms featuring the viral transcriptional response to UV irradiation are known, the host and/or viral molecular components involved in this process as well as the chronological transcriptional activation have not been elucidated yet. Conversely, a fine-tuned transcriptional regulation circuit that ruled the maintenance of the SSV1 carrier state has been recently unveiled [33,54]. The discovery of the T_lys_ transcript led to the evidence that its encoded transcriptional factor F55 is the main viral player in the maintenance of the lysogeny of SSV1 and in regulating the transition from the carrier to the UV-inducible state. Intracellular concentration of F55 was regulated by transcriptional activation/repression as well as by a post-transcriptional mechanism of RNA degradation, as observed in the aftermath of UV irradiation [33,54]. A detailed description of F55’s role is included in its proteomic paragraph. It remains to be established whether other regulation circuits might be involved in the transcriptional activation of the UV-inducible promoters.

## 4. Proteomic Analysis

As for most of the spindle-shaped archaeal hyperthermophilic fuselloviruses, for a long time, putative genes of SSV1 did not match with any genes in public databases [55,56]. Consequently, it has been challenging to infer, through homology analysis, the role of SSV1 proteins in its life cycle, although some structural and bioinformatics investigations have provided reasonable functional predictions for several other SSV1 proteins [44]. The function of some SSV1 structural proteins (VP1, VP2, and VP3) was discovered in the past [24]. Conversely, all the constituents of SSV1 virus particles (virions) remained only partially described until 2015, when Quemin et al. carried out a thorough biochemical characterization of SSV1 virions [36]. Specifically, they identified a fourth (besides VP1, VP2, and VP3) virus-encoded structural protein, i.e., VP4 (formerly known as C792). Thus, the bulk of SSV1 virions is made up of multiple copies of VP1, VP2, and VP3. Terminal virion fibers involved in virion aggregation [36] and in host recognition and attachment [52] are instead constituted by oligomers of VP4. Moreover, by means of a glycoprotein-specific stain, it was shown that VP1, VP3, and VP4 are glycosylated at multiple sites. As for other proteins from *Sulfolobales* [57], SSV1 structural proteins are glycosylated at asparagine residues within the consensus motif N-X-S/T (where X is any amino acid except proline). Both VP1 and VP3 possess two such motifs, whilst VP4 contains 20 consensus motifs that could potentially undergo glycosylation [36].

Furthermore, the same authors discovered that a DNA-binding protein of cellular origin (Sso7d) is part of the virion structure. Since the host-encoded Sso7d is a member of the Sul7d protein family, which are responsible for chromosome organization in *Saccharolobus*, it has been speculated that Sso7d could act during virion packaging to organize and condense the viral genome. Whereas VP1 and VP3 paralogous have homologs in all known spindle-shaped viruses, VP2 has been found to be encoded only by 10 fuselloviruses for which complete genome sequences are available (Figure 2, shell genome, OG0000046). Moreover, VP2 is not specific to fuselloviruses; indeed, homologs of this protein are encoded by unrelated archaeal and bacterial viruses. In accordance with the scarce conservation among fuselloviruses, deletion of the *vp2* gene did not affect virion assembly or infectivity, thus suggesting that the protein VP2 is dispensable at least under laboratory growth conditions. In line with its scattered phyletic distribution and dispensability, it has been hypothesized that the *vp2* gene was acquired relatively late in the history of fuselloviruses from a different group of viruses [36].

So far, among the 35 predicted SSV1-encoded proteins, structural data are available only for very few proteins; the last SSV1 protein structure (A100) was released in 2014. Atomic resolution structures are available for five putative proteins with unknown functions (i.e., B129, D63, F93, F112, and A100), as well as for the integrase (D335) [55,58,59]. Nevertheless, the lack of functional characterization makes it difficult to assign a defined function to these proteins. For instance, a reasonable prediction of D63 function was limited by the fact that its secondary structural motifs are widespread in many proteins covering a broad range of functions. Only the similarity of its three-dimensional structure with the bacterial adaptor protein repressor of primer (ROP) suggested a possible role of D63 in the regulation of the SSV1 episomal copy number [55,60]. Conversely, structural analyses of the integrase have shown a similar core fold to both bacterial and eukaryal recombinases, except for the lack of the typical helix corresponding to αI of Cre. Therefore, this enzyme was unequivocally assigned to the type I tyrosine recombinase family. Moreover, functional analyses have shown that it possesses an N-terminal domain responsible for protein dimerization and a C-terminal domain capable of DNA cleavage and ligation [59].

Except for few examples, most of SSV1 proteins remain uncharacterized and/or lack an assigned function. In the attempt to address this issue, Iverson et al. designed a genetic approach that included both specific and random mutagenesis to introduce mutations in all SSV1 ORFs [16]. Although this approach did not clarify the exact role of SSV1 proteins, it helped to understand at least which proteins were essential for the infectivity of the SSV1 virions. In particular, by means of long inverse PCR (LIPCR) and transposon mutagenesis, the authors constructed 78 variants of the SSV1 genomes harboring mutations in each of the 35 ORFs. All these virus mutants were tested for their ability to infect the permissive host *S. solfataricus*. By doing so, the authors have shown that nearly half of the SSV1 genes (16 out of 35 ORFs) were dispensable for the SSV1 virions infectivity and that most of essential genes are highly conserved within the *Fuselloviridae* family and constitute the so-called “fusellovirus core” [34]. Whereas almost the entire T5 early transcript was shown to be dispensable (7 out of 10 ORFs), many proteins encoded by the T6 early transcript were essential, which is in line with the abundance of well-conserved fusellovirus core genes in this transcript. Most of core genes were intolerant to insertions and/or deletions, thus indicating an essential role of the encoded proteins for the SSV1 infection cycle. A surprising exception was represented by the minor capsid gene *vp3* that, despite belonging to the fusellovirus core genes, appears to be dispensable for SSV1 infectivity. However, mutations of this gene resulted in an abnormal morphology with virions longer and thinner than the wild type [16]. Even though deletion of the integrase gene led to a virus mutant (SSV1-Δd335) that could not infect the strain used in the study by Iverson and colleagues, this core gene was dispensable at least in some other hosts (i.e., *S. solfataricus* strain P2 and Gθ) [61]. These apparently contradictory results might have been due to the presence of a host-encoded integrase that, in some *Saccharolobus* strains, could be active on SSV1 DNA [16,34]. This study also showed that *b49* and *f55* can be deleted from SSV1 without loss of infectivity. However, the spot-on-lawn or halo assays performed in this study did not allow for the quantification of virion number in the culture supernatant; therefore, it cannot be completely excluded that the deletion of *f55* caused an alteration of SSV1 titer during lysogeny (carrier state) without interfering with the infectivity of SSV1 virions.

**Figure 5:**
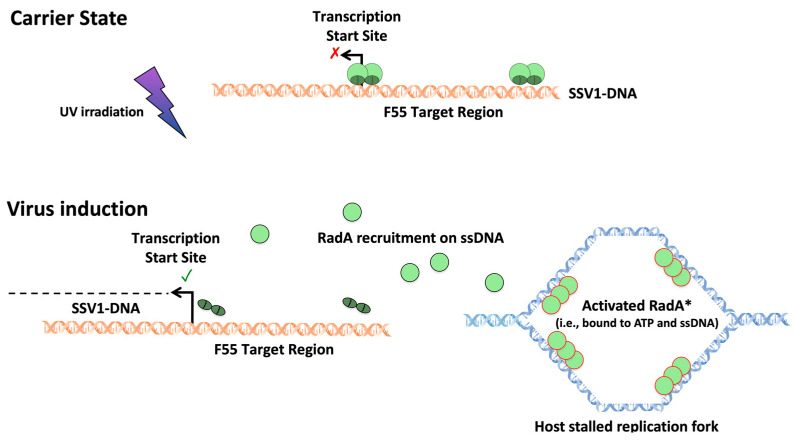
**SSV1 carrier-to-induction state transition stimulated by host DNA damage.** In the carrier state, multimers of F55 are bound to all the target sites on the SSV1 genome, thus repressing transcription (represented as red cross) of the viral early genes. The interaction of F55 to the virus DNA is stabilized by the co-presence of the host encoded RadA protein. UV irradiation damages the host DNA, which in turn causes the accumulation of stalled replication forks (ssDNA) in regions of the host genome where DNA contains unrepaired lesions or lesions that are undergoing repair. This event fosters the recruitment of RadA to ssDNA regions (indicated as activated RadA*), where it forms nucleoprotein filaments. In turn, this event results in the progressive release of RadA from the virus DNA and, later on, in the dissociation of F55 from its target sites with the consequent activation of the temporary coordinated transcription pattern of SSV1. The model is adapted from Fusco et al., 2022 [45].

Even though SSV1 has been the most studied fusellovirus in the past decades, the molecular mechanism involved in the transition from carrier state to transcription/replication induction remained unclarified for long time. It was only in 2013 that the discovery of an additional and previously unidentified viral transcript (i.e., T_lys_) allowed the shedding of light on how this virus regulates its infection cycle [33]. This initial in vitro study demonstrated that T_lys_ encodes for a 55-amino-acid protein (F55). The recombinant F55 was shown to specifically bind the SSV1 DNA at an 11 nt binding sequence (consensus sequence: 5′-ATAGATAGAGT-3′), which is present in several copies in the promoter region of T_5_, T_6_, and T_ind_, as well of its own transcript (i.e., T_lys_). In order to elucidate the regulatory role of F55, more analyses were carried out to measure the level of F55 protein (before and after UV light exposure) in infected cells of *S. solfataricus* to confirm its interaction in vivo with the promoters of T_5_, T_6_, T_ind_, and T_lys_ [54]. Upon UV irradiation, it was shown that F55 first dissociates from target sequences in the T_ind_ promoter (2 h post treatment) and later from those in the T_5_ and T_6_ promoters (4 h post-treatment). The progressive release of this transcription regulator from its binding sites is in agreement with the differential (in vitro measured) affinity of F55 for these sequences (*K_d_* = T_5_ ≃ T_6_ < T_ind_) [33]. Given that T_ind_ and T_5_/T_6_ are transcribed after 2 h and 4 h after UV irradiation, respectively, the release of F55 from the promoter regions of these transcripts might be the molecular event that activates their transcription [54]. Nonetheless, it was still not clear how SSV1 could “sense” the host cell damage caused by the environmental stress (i.e., the UV irradiation). For this reason, a variant of the electrophoretic mobility shift assay (EMSA) coupled to Western blot and mass spectrometry analyses was used to unravel the protein-protein interaction network (interactome) between SSV1 and its host *S. solfataricus* [45]. Intriguingly, among F55 interactors, the enzyme RadA (the archaeal homolog of the bacterial RecA) was identified, which is essential for genome stability. Specifically, RadA mediates non-specific DNA repair by binding single-stranded DNA (ssDNA) at collapsed replication forks and catalyzing homologous base pairing and strand exchange [62]. The interaction of a viral regulator with a host protein involved in DNA repair it is not unusual. For instance, it is used by the well-characterized bacteriophage λ to regulate the switch from lysogenic to lytic development. Interestingly, it was shown that in SSV1-infected cells, RadA binds to F55 to generate a stable tripartite RadA–F55–dsDNA complex that can be destabilized by the formation of stalled DNA replication forks (Figure 5). Because of the RadA recruitment at ssDNA regions, the interaction of F55 with DNA at its target sites was destabilized, thus leading to the dissociation of this transcription repressor from promoters of T_ind_, T_5_, and T_6_ that, in turn, activates their transcription. Even though it has not been unequivocally demonstrated that the interaction between F55 and its operators is destabilized because of RadA detachment, it was speculated that the interaction between F55 and RadA represents the molecular sensor that SSV1 uses to control host genome integrity and, in turn, to regulate its own infection cycle [45].

## 5. Host–Virus Interaction

SSV1 was first identified as a plasmid replicating in *S. shibatae* B12 [63]. Then, the viral nature of SSV1 was demonstrated when spindle-shaped virus-like particles present in the supernatant of the culture were found to infect *S. solfataricus*. The conditions to obtain the highest virus titer under lab conditions have been studied, and a protocol for the UV induction of SSV1 replication based on the combination of several parameters is available [15]. Since the physiology of the SSV1 life cycle was identical in *S. shibatae* and *S. solfataricus*, the latter has been widely used as a host model for molecular, genetic, and physiological studies on SSV1 [63]. It integrates into the host genome at the arginyl-tRNA gene, which is maintained intact after integration. Moreover, the integrase that promotes site-specific integration has been biochemically characterized [20,61]. As a unique feature of the host–virus interaction of an UV-inducible virus, the release of SSV1 viral particles does not cause lysis of host membrane, and cells recover their growth rate as well as their carrier state within a few hours [20].

### 5.1. Transcriptome Analysis of S. Solfataricus

A transcriptomic analysis has been performed on *S. solfataricus* lysogens to study the variation of host/virus gene expression upon UV irradiation [23]. It was observed that the host response included down- or upregulation of several genes in comparison to the uninfected cells, suggesting a major sensitivity of SSV1 lysogens to UV irradiation. Only a few genes exhibited a differential transcriptional variation specifically related to the presence of the SSV1 virus; however, the strength and speed of the general UV response was significantly different in uninfected and infected cells, indicating that the presence of the virus and/or of some viral components increased the host sensitive to the UV-induced DNA damage. Among the very few genes that showed a differential response, worth noting are those encoding the two subunits of topoisomerase VI that might be involved in DNA supercoiling during DNA replication [25]. Interestingly, the temporal concurrence of the upregulation of chromosomal genes and of the early genes of SSV1 indicates a potential virus–host co-regulation. Differently from what was seen for infected cells upon exposure to UV irradiation, the host metabolism and growth is not visibly impaired by the presence of the SSV1 virus in the absence of UV stimulus. Indeed, about 30 genes were found to be up- or downregulated in stable SSV1 lysogens, confirming that SSV1 had only a limited effect on the host gene expression in the carrier state [23]. The absence of a strong host response hinted to a harmonic host–virus co-existence in the absence of UV irradiation. Accordingly, SSV1 is a temperate virus able to self-regulate its own replication through the pleiotropic effect of F55, which represses the expression of the UV-inducible as well as of the early genes (see above) [33,54]. Whereas SSV1 did not induce major changes on the host gene expression, SSV2, which is closely related to SSV1 in terms of morphology, genome organization, and gene synteny, elicited a strong host response (162 up- and downregulated genes vs. 30 SSV1 genes), which includes transcriptional activation of CRISPR loci and *cas* genes. This response has been attributed to the difference in life cycles between SSV1 and SSV2, since only the latter exhibits a strong physiological induction during the host growth. This led to the activation of the CRISPR response to control the SSV2 induction and to safeguard host genome integrity through deletion of self-targeting spacers [23]. Interestingly, it was observed that in lysogens containing both SSV1 and SSV2, the presence of the former quenched the effect of SSV2 infection on the host gene expression, and specifically, no differential expression has been observed for genes and clusters of the CRISPR-Cas system. It is plausible that transcription factors and/or other molecular components encoded by SSV1 are involved in silencing the CRISPR-Cas response in the SSV1-SSV2-infected strain [23]. The harmonic coexistence between SSV1 and its host is possible thanks also to the virus egress strategy that apparently do not cause cell lysis [20,64]. The morphogenesis of the SSV1 virus particles has been extensively studied in the natural host *S. shibatae*, demonstrating that SSV1 assembly and egress are concomitant and occur at the cellular cytoplasmic membrane via a budding-like process followed by the maturation into characteristic spindle-shaped virions. The proposed model explains why SSV1 is encased in lipid-containing envelopes similarly to the enveloped eukaryotic viruses that exploit the same egress strategy. Less understood is how SSV1 gains access to the *Saccharolobus* host cells. Previous studies indicated that the S-layer in *S. solfataricus* P1 is required for SSV1 adsorption since S-layer depleted cells are less susceptible to SSV1 infection [65]. To sum up, the coexistence of the virus with the natural and *S. solfataricus* lysogens is characterized by (i) maintenance of low copy number in the absence of UV irradiation, (ii) negligible effect on the host metabolism, and (iii) the non-lytic egress of SSV1 [64,66]. All together, these features allowed for a close co-evolution of this virus with its natural host.

### 5.2. Regulation of CRISPR-Cas System during the Infection

Compared to other SSV representatives, SSV1 seems to have the narrowest host range, infecting only three *S. solfataricus* strains and a single *Saccharobus* sp. isolated from Lassen Volcanic National Park [52]. Records of previous infection of *S. tokodaii* have been revealed by the analysis of the CRISPR spacers containing a 41 bp long sequence matching SSV1. However, *S. tokodaii* turned out to be resistant to the infection of all the SSVs viruses, including SSV1. This suggests that this host has developed efficient strategies to circumvent the SSVs virus entry/infection. Similarly, *S. acidocaldarius* is also insensitive to SSV infection, although no CRISPR matches toward these viruses have been found [12]. It is now widely accepted that CRISPR-Cas defense systems might have, to a large extent, shaped the evolution of the viral genomes. Nevertheless, the above-mentioned pieces of evidence suggest that the infectivity of SSVs toward their potential host is related not only to the CRISPR system but also to complex not yet elucidated mechanisms, including regulation of adsorption, other aspects of DNA entry, genome replication, transcription, translation, assembly, or virus release. At present, there is no explanation for the narrow host range of SSV1. In general, SSV infectivity and *Saccharolobus* susceptibility are independent of the geographic regions from which the hosts and viruses were isolated [12]. It is tempting to speculate that the UV-inducible nature of SSV1, which is unique among all the known crenarchaeal viruses, along with the lack of evolutionary connection with other SSVs, have influenced the virus physiology and in turn the “coevolutionary arms race” of the host–virus interaction [67].

## 6. Conclusions

Viruses represent one of the major agents of evolution by virtue of their capacity to operate exchange of genetic material (horizontal gene transfer, HGT) and to move actively among biomes [68]. In particular, archaeal virology and metaviromics are opening the way to answer questions regarding the origin of viruses and their position in the global virosphere [68]. Furthermore, the study of virus–host model systems, such as SSV1 and *Saccharolobus* spp., is functional to shedding light on the evolution and on the regulatory mechanisms of viruses and their relative hosts [9,10,24,28,69,70,71,72]. Comprehensive studies on SSV1 have definitely contributed to elucidating fundamental molecular mechanisms in *Saccharolobus* hosts as well as to set up valuable genetic tools [21,73,74,75]. For instance, these systems have been successfully used to unveil the role of SSV1 proteins in the infection cycle [16] and to express heterologous enzymes [18,75]. Finally, a wide variety of biotechnological applications such as medicine, agriculture, pharmacology, cosmetics, electronics, nanotechnology, and environmental safeguarding locate extremophilic microorganisms [76,77,78,79,80,81,82,83,84,85,86] and their viruses as valuable tools [2,87,88].

## Figures and Tables

**Table 1:** Putative transcriptional regulators in SSV1.

Protein	Annotation	Core-Gene	Distribution in Crenarchaeal Viruses	Reference
F93	winged helix-turn-helix (wHTH)	N	*Fuselloviridae, Portogloboviridae, Guttaviridae*	[48]
F112	winged helix-turn-helix (wHTH)	N	unique to SSV1	[43]
B115	winged helix-turn-helix (wHTH)	Y	*Fuselloviridae*	
F55	ribbon-helix-helix (RHH)	N	unique to SSV1	[33,45]
C80	ribbon-helix-helix (RHH)	Y	*Lipothrixviridae, Bicaudaviridae, Rudiviridae, Spiraviridae*	
E51	ribbon-helix-helix (RHH)	N	*Fuselloviridae*	
A79	zinc-finger	Y	*Fuselloviridae, Rudiviridae, Portogloboviridae, Ovaloviridae*	
B129	zinc-finger	Y	*Fuselloviridae*	[44]
A45	zinc-finger	N	*Fuselloviridae, Lipothrixviridae*	
C102	zinc-finger, putative membrane protein	Y	*Fuselloviridae, Guttaviridae*	
A100	Pur-alpha repeat	Y	*Fuselloviridae, Rudiviridae, Portogloboviridae*	

## Supplementary Materials

The following supporting information can be downloaded at https://www.mdpi.com/article/10.3390/v14102082/s1, File S1: Genome accession numbers of the members of the Fuselloviridae family (15 January 2022); File S2: Presence-absence matrix of groups of orthologous proteins in 49 members of the Fuselloviridae; File S3: Accession numbers of proteins of the different orthologous groups of the Fuselloviridae; File S4: Annotation of fuselloviral capsid proteins VP1 and VP3 using the prokaryotic viral ortholog groups (pVOGs) database.
